# Vibrational Analysis of Thermoelastic Beams on Dual-Parameter Foundations via the Fractional Three-Phase-Lag Approach

**DOI:** 10.3390/mi17020241

**Published:** 2026-02-12

**Authors:** Adam Zakria, Ahmed Yahya, Ibrahim-Elkhalil Ahmed, Ibrahim Omer Ahmed, Abdelgabar Adam Hassan, Muntasir Suhail, Eshraga Salih

**Affiliations:** 1 Department of Mathematics, College of Science, Jouf University, Sakaka 72388, Saudi Arabia; ielkhail@ju.edu.sa (I.-E.A.); iobudawe@ju.edu.sa (I.O.A.); aahassan@ju.edu.sa (A.A.H.); 2 Department of Mathematics, College of Science, Qassim University, Buraydah 52571, Saudi Arabia; m.suhail@qu.edu.sa (M.S.); e.salih@qu.edu.sa (E.S.)

**Keywords:** fractional three phase lag, thermoelastic, microbeam, vibration, foundation

## Abstract

This study introduces a unified analytical framework for investigating the transient thermoelastic vibration of a micro-scale beam resting on dual-parameter foundations. We apply the fractional three-phase-lag (FTPL) generalized thermoelastic model to accurately characterize scale-dependent coupled thermal and elastic responses by incorporating complex thermal relaxation effects through the fractional derivative order. By employing the Laplace transform technique and its numerical inversion, we derive the coupled distributions of temperature, displacement, bending moment, and deflection within the beams. A comprehensive parametric analysis is conducted to quantify the distinct influence of the fractional factor and the foundation’s shear and stiffness parameters on the beam’s dynamic stability and propagation characteristics. The calculated results are systematically compared with established classical theories to validate the model’s robustness while simultaneously demonstrating the enhanced predictive capacity of the (FTPL) approach, particularly for characterizing thermal wave dispersion at the micro-scale. This research provides critical design criteria for advanced micro-electromechanical systems (MEMSs) where foundation stiffness and thermal inertial effects are intrinsically linked, offering novel insights into the tailored design of microstructural components.

## 1. Introduction

In the early stages of design, beams models were commonly used because they provided fundamental insights into the behavior of structures. These models are also effective for verifying computational solutions. Various microbeam models have been developed with varying accuracies depending on the underlying assumption. The Euler–Bernoulli beam model is among the most important models. Microbeams are often integrated into microstructured systems and devices, such as sensors and actuators. The study of the vibration characteristics of microbeams has attracted significant attention owing to their wide application in MEMS and NEMS systems [1,2,3,4,5].

Recent studies have addressed the thermoelastic behavior of microbeam vibrations propped by a Pasternak foundation. Notably, researchers, including those mentioned in [6,7,8,9,10,11], are at the forefront of these studies. Not only did Ansari and Hosseinzadeh [12] and Attia and Salwa [13] expand our understanding, but they also paved the way for many important publications. These researchers explored the vibration characteristics of a micro-scale beam subjected to a moving mass, thereby providing fundamental insights into the design and construction of structures. Furthermore, critical research has focused on the behavior of beams supported by dual-modulus elastic foundations, which have distinct properties relevant to design and related industries. This research, which is the subject of numerous theoretical studies [14,15,16], is crucial because many structures and systems can be classified as foundation-supported beam structures. Geotechnical engineering plays a fundamental role in the study of soil-reinforced foundations. The multidisciplinary nature of this study is evident, as it draws on knowledge from fields such as structural engineering, geotechnical engineering, and materials science. Modeling soil responses to external forces is essential for providing a consistent and manageable approach for describing the complex stress-strain characteristics of most natural soils. The results of this study are practical because they demonstrate that increasing the Pasternak factor reduces the dynamic response of the micro-scale beam, resulting in a significantly faster beam setup procedure.

In addition, Adam and Abouelregal [17], Saadatnia et al. [18], and Hettiny [19] explored the characteristics of a foundational model that integrates a two-phase viscous Pasternak approach with a standard method for analyzing the bending of beams on an elastic basis. Lord and Shulman [20] and Chen et al. [21] investigated the dynamic stiffness matrix of beams resting on elastic foundations and exposed them to a harmonic moving load. Kargarnovin and Younsian [22] examined the dynamic behavior of beams supported by generalized Pasternak parameters under a randomly distributed harmonic moving load. Additionally, the Pasternak foundation is an essential tool for studying soil–structure interactions in various engineering contexts. Its ability to consider both compressive and shear stresses in soil makes it particularly useful for analyzing the behavior of beams and slabs on soil foundations, providing more accurate results than simpler models [23,24,25].

The traditional model of heat conduction, grounded in Fourier’s law, has been extensively utilized to explain thermal transport in solids. For example, Lord and Shulman [20] improved the Fourier model to incorporate a single-phase element, which accounts for the effects on a very short timescale. Biot and Willis [26] established a classical thermoelasticity (CTE) model that offers a basic understanding of the material behavior under thermodynamic and elastic conditions. However, recent advances in nanotechnology and materials science have highlighted the limitations of this traditional method, particularly when applied to nano- and micro-scale systems. Consequently, several non-instantaneous heat conduction models have been developed to overcome these limitations. An important alternative is the dual-phase-lag (DPL) model, which has been used in various contexts, such as ultrafast laser heating and heat conduction in carbon nanotubes, as pointed out by Shomali et al. [27]. An additional method is the three-phase-lag (TPL) model, which builds on the DPL model by presenting three distinct phase lags for heat flow, temperature variation, and thermal displacement variation, as discovered by Abouelregal [28]. These models strive to capture nonclassical heat transfer phenomena that go beyond the traditional Fourier framework, including ballistic transport, phonon hydrodynamics, and coherent phonon transport, as considered by Su et al. [29].

In recent decades, classical thermoelasticity has been used to accurately describe material behavior at micro- and nanoscales. This prompted the development of nonlocal thermoelasticity, a generalized theory that explains these size-dependent phenomena. Combining nonlocal elasticity with generalized thermoelasticity has led to the development of comprehensive nonlocal thermoelastic models. These models are essential for the accurate analysis of the thermomechanical behavior of a variety of advanced materials and structures, including thin films, nanowires, micro-scale beams, and functionally graded materials, which are increasingly used in micro- and nanoelectromechanical systems (MEMSs/NEMSs) [30,31,32]. By incorporating nonlocal impacts, these theories provide more accurate predictions of the stress, strain, temperature, and deformation fields, which are critical for the reliable design and performance of miniature devices where surface and microstructural effects are important. Recent studies [33,34,35] in this area have addressed specific applications such as the unified generalized thermoelastic formula for evaluating thermoelastic damping in micromechanical resonators. This study strengthens the existing literature on analytical solutions by providing a comprehensive investigation of thermoelastic vibrations in microbeams resting on dual-parameter foundations (DPF). In particular, this work builds upon and expands the analytical techniques utilized in related complex flow and symmetry problems, such as the implementation of Lie symmetry analysis and conservation laws to determine wave solutions for stochastic potential-(KdV) equations [36], and the derivation of precise mathematical models for the unsteady rotational flow of fractional-order non-Newtonian fluids under boundary shear stress conditions [37]. By combining these advanced analytical methodologies with generalized fractional three-phase-lag (FTPL) thermoelasticity model, this study provides crucial design criteria and new insights into the dynamic stability of MEMS/NEMS resonators where foundation stiffness and thermal inertial effects are closely related.

Based on these basic models, this study presents an advanced fractional three-phase-lag (TPL) model to address the limitations of previous approaches. The fractional three-phase-lag (TPL) model has emerged as an influential method for examining the thermoelastic interactions in a variety of materials and structures. When utilized for microbeam resonators, the TPL model offers significant insights into thermoelastic damping and its impact on resonator functionality, as examined by Kumar et al. [38]. The incorporation of fractional calculus into thermoelastic theories by Abouelregal et al. [39] resulted in more detailed models that effectively captured intricate heat transfer processes and mechanical interactions. These models have been effectively applied in previous studies [40,41] across different scenarios, such as functionally graded materials and nanobeams. The fractional TPL model provides enhanced approximations for heat flux, temperature gradients, and thermal displacement, integrating nonlocal effects and higher-order time derivatives, as reported by Abouelregal [42]. The mathematical significance of the fractional-order method is further underscored by recent analytical advancements in heat transfer and fluid mechanics. For instance, the Prabhakar fractional derivative (PFD) approach has been successfully employed to derive generalized Mittag-Leffler kernel solutions for complex phenomena like free convection in Maxwell fluids [43]. These sophisticated operators provide a versatile framework for describing anomalous transport processes that standard integer-order derivatives cannot capture. By aligning the current fractional three-phase-lag (FTPL) model with such rigorous developments, this study establishes a robust foundation for capturing the intricate nonlocal and memory-dependent thermoelastic interactions inherent in micro-scale structures. In the realm of microbeam vibrations on Pasternak parameters, the fractional TPL model can offer a more precise depiction of the thermoelastic behavior. This method enables the investigation of size-dependent influences, thermal influences, and the influence of basis parameters on the dynamic response of the small-scale beam. By combining the fractional TPL model with Pasternak’s rule, scientists can gain a deeper understanding of complex behavior, including thermal and mechanical aspects in microstructures, which could lead to improvements in the design of micro-electromechanical systems (MEMSs) and other microdevices. The practical effectiveness of fractional-order models is becoming increasingly evident across various scientific-fields due to their superior predictive capabilities for complex and time-varying systems. For example, fractional-order lag models in epidemiology effectively capture the effects of memory and genetic properties [44,45]. Adopting a similar framework for resonators in micro/nanoelectromechanical systems provides a rationale for using fractional derivatives instead of conventional ones. This approach offers a more realistic characterization of unconventional heat transfer and complex thermoelastic interactions in microstructures, providing crucial design criteria and new insights into the dynamic stability of micro-components.

The main innovation of this manuscript is the formulation and utilization of a novel fractional TPL model based on a two-parameter elastic foundation for the vibration analysis of generalized thermoelastic beams. Originating from a recent investigation into three-phase-lag (TPL) heat conduction with fractional configurations and operators, this model offers a novel perspective on the complex interaction between the thermal and mechanical properties of materials with intricate microstructures compared with traditional thermoelastic models, such as classical theories, which often fail to accurately predict thermal and mechanical responses at micro-scales or in materials exhibiting nonlocal effects and memory effects. These properly arranged models have difficulty describing anomalous diffusive or viscoelastic behaviors inherent in many advanced materials and micro/nanostructures. Additionally, this study creatively applied this model to examine the heat transfer in beams and conducted a comparative analysis of various thermoelastic models within this framework. Finally, it meticulously observes and graphically represents the impact of the fractional-order parameter (α), two-parameter elastic foundation, different thermoelastic models, and other physical field components on the vibration of the analyzed microbeam fields through visual comparisons.

## 2. Basic Equations of the Methodology

**Figure 1:**
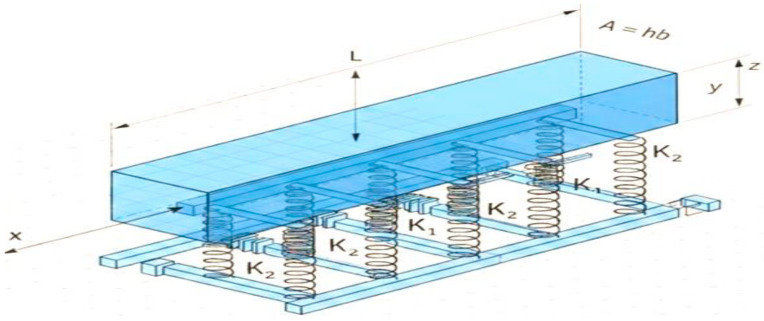
Simple diagram of the mechanical model of the beam.

In this section, a rectangular beam (Figure 1) with length (L: 0 ≤ x ≤ L) and thickness (h:−h/2 ≤ z ≤ h/2) is selected, and the cross-sectional area is calculated as (A=hb). The beam’s axis is oriented along the (x) coordinate, while the (y) and (z) coordinates represent the width and thickness, respectively. This beam is constructed from a homogeneous, isotropic, and linearly elastic material, defined by its modulus of elasticity (*E*) and Poisson’s ratio (ν). This was supported by a homogeneous elastic soil basis, which was modeled using three parameters. The basis model includes linear Winkler foundations $\left(K_{1}\right)$ and shear foundations $\left(K_{2}\right)$.

In response to the limitations of the Winkler model, enhanced theories have been presented that incorporate different contact types, such as beams and shear layers, along Winkler springs [46]. These theories aim to create a straightforward and practical model for depicting the basic medium. Models that consider these two factors are more precise than those that consider a single parameter. Notably, if the second parameter is disregarded, the mechanical modeling of the foundation simplifies the Winkler formula. The basis medium is assumed to be linear, homogeneous, and isotropic. Hetenyi [19] and Kerr [46] studied a beam propped by a Pasternak parameter, which included both spring and shear stiffness, and analyzed the interaction stress of the two-parameter basis when imperiled to a distributed load, as follows:

(1) $$P\left(x,t\right)=K_{1}w\left(x,t\right)-K_{2}\frac{\partial^{2}w\left(x,t\right)}{\partial x^{2}}.\;$$

where w is the lateral deflection and P(x,t) is the basic reaction per unit area.

The components of the displacement vector constructed based on Euler–Bernoulli beam theory [47,48] are:

(2) $$u=-z\frac{\partial w}{\partial x},v=0,\;w\left(x,y,z,t\right)=w\left(x,t\right).\;$$

We can apply Equation (2) to the 1D case. Thus, the constitutive equation is expressed as follows:

(3) $$\sigma_{x}-e_{0}\frac{\partial^{2}\sigma_{x}}{\partial x^{2}}=-E\left[\frac{\partial^{2}w}{\partial x^{2}}+\alpha_{T}\theta \right].$$

where $\sigma_{x}$ is the nonlocal axial stress, $e_{0}$ is the nonlocal parameter, E is the modulus of elasticity, $\alpha_{T}=\alpha_{t}/(1-2\nu )$ is the type of nonlocal elasticity, and θ is the change in temperature.

We can obtain the moment M from Equation (3), as follows:

(4) $$M\left(x,t\right)=e_{0}\frac{\partial^{2}M}{\partial x^{2}}-IE\left[\frac{\partial^{2}w}{\partial x^{2}}+\alpha_{T}M_{T}\right].$$

In this context, when ($\alpha_{T}M_{T}$) is multiplied by the flexural rigidity of the beam (IE), the result is a genuine thermal moment consistent with the geometry and material properties of the beam. It represents an equivalent thermal “curvature” that accounts for the beam’s cross-section [49,50,51] and $\left(M_{T}\right)$ is given by:

(5) $$M_{T}=\frac{12}{h^{3}}\int_{-h/2}^{h/2}\theta \left(x,z,t\right)zdz.\;$$

The motion equation for the transverse response of the microbeam is represented as follows:

(6) $$\frac{\partial^{2}M}{\partial x^{2}}=P\left(x,t\right)+\rho A\frac{\partial^{2}w}{\partial t^{2}},$$

where (ρ) represents the density of the microbeam material and (A) represents the cross-sectional area of the microbeam.

Furthermore, as derived from Equation (4) and referenced in [25], the moment of the microbeam can be accurately formulated as follows:

(7) $$M\left(x,t\right)=\rho A\frac{\partial^{2}w}{\partial t^{2}}+K_{1}w\left(x,t\right)-\left(IE+K_{2}\right)\;\frac{\partial^{2}w\left(x,t\right)}{\partial x^{2}}-\alpha_{T}M_{T}.$$

According to a previous study [17] and using Equations (6) and (7), we obtain the equation of motion for the microbeam, as follows:

(8) $$\frac{\partial^{4}w}{\partial x^{4}}-\frac{{e_{0}K}_{1}+K_{2}}{IE+e_{0}K_{2}}\frac{\partial^{2}w}{\partial x^{2}}+\frac{\rho A}{IE+e_{0}K_{2}}\frac{\partial^{2}}{\partial t^{2}}\left(w-e_{0}\frac{\partial^{2}w}{\partial x^{2}}\right)+\frac{K_{1}}{IE+e_{0}K_{2}}w+\frac{\alpha_{T}}{IE+e_{0}K_{2}}\frac{\partial^{2}M_{T}}{\partial x^{2}}=0.$$

Equation (8) delineates a complex PDE that governs the transverse vibration of a micro-scale beam by integrating the mechanical, thermal, and foundation impacts. Each term in this equation represents a distinct physical phenomenon that contributes to the dynamic response of the beam [52,53].

Lord and Shulman [20] described the heat conduction equation within the framework of generalized thermoelasticity theory via the following formula:

(9) $$\left(1+\tau_{q}\frac{\partial }{\partial t}\right)\left(\frac{\partial^{2}T}{\partial x^{2}}+\frac{\partial^{2}\theta }{\partial z^{2}}\right)=K\left(1+\tau_{\theta }\frac{\partial }{\partial t}\right)\left[\rho C_{E}\frac{\partial \theta }{\partial t}+\gamma T_{0}\frac{\partial e}{\partial t}\right].$$

The coefficients $\tau_{\theta }$ and $\tau_{q}$ are properties of generalized thermoelastic theories, which allow a finite speed of heat propagation.

## 3. TPL Fractional-Order Heat Conduction Equation

The fundamental principle governing heat transfer is encapsulated in Fourier’s rule, establishing a direct association among the heat flow (q) and temperature variation (∇T), with the thermal conductivity (K) acting as the mediating parameter. This relationship can be expressed as follows:

(10) $$\vec{q}\left(Z,t\right)+K\nabla T\left(Z,t\right)=0.$$

As explained in [20], this equation demonstrates that heat transfer occurs from regions of higher temperatures to those of lower temperatures; Green and Naghdi [54,55] described the general form of Equation (10) as follows, where K* demonstrations the characteristic material constant of the theory

(11) $$\vec{q}\left(Z,t\right)+K\nabla T\left(Z,t\right)+K^{*}\nabla \theta \left(Z,t\right)=0.$$

In view of the above developments in heat conduction models, Choudhuri [56] proposed the TPL model by adding a new phase lag $\tau_{3}$ in addition to the classical phase lags $\tau_{1}$ and $\tau_{2}$ for $\vec{q}$ and ∇T for the ∇θ, as follows:

(12) $$\vec{q}\left(Z,t+\tau_{1}\right)+K\nabla T\left(Z,t+\tau_{2}\right)+K^{*}\nabla \theta \left(Z,t+\tau_{3}\right)=0,$$

where $0<\tau_{3}<\tau_{2}<\tau_{1}$.

If divergence is applied to both sides of Equation (12), we obtain

(13) $$\nabla \cdot \vec{q}\left(Z,t+\tau_{1}\right)+K\nabla^{2}T\left(Z,t+\tau_{2}\right)+K^{*}\nabla^{2}\theta \left(Z,t+\tau_{3}\right)=0.$$

The nonlocal generalized form of Equation (13) was obtained through series expansion based on the formal theory of fractional thermal hardness, as explained by Sherif et al. [26]. This expansion results in the following constitutive relationship:

(14) $$T_{1}\nabla \cdot \vec{q}=-KT_{2}\nabla^{2}T-K^{*}T_{3}\nabla^{2}\theta .$$

The Biot’s energy equation [57], expressed in terms of the heat flux vector ($\vec{q}$), is denoted as follows:

(15) $$\frac{\partial }{\partial t}\left(\rho C_{E}T+\gamma T_{0}e\right)-Q=-\nabla \cdot \vec{q},$$

By incorporating the nonlocal generalized three-phase-lag constitutive relation (Equation (14)) into the energy equation (Equation (15)), the resultant fractional three-phase-lag heat conduction equation for the theory under deliberation is derived as

(16) $$T_{1}\left(\frac{\partial }{\partial t}\left(\rho C_{E}T+\gamma T_{0}e\right)-Q\right)=\;\;KT_{2}\nabla^{2}T+K^{*}T_{3}\nabla^{2}\theta .$$

The constitutive relationship among temperature due to conduction (T) and absolute temperature (ϑ) is as follows:

(17) $$T=\vartheta +e\nabla^{2}T.$$

By substituting Equation (17) into the fractional three-phase-lag heat conduction equation (Equation (16)) and disregarding terms including Laplace operators of order greater than two, we derive the following equation, which facilitates the investigation of the coupled impacts of thermodynamic and conductive temperatures:

(18) $$T_{1}\left(\frac{\partial }{\partial t}\left(\rho C_{E}\vartheta +\gamma T_{0}e\right)-Q\right)=\;KT_{2}\nabla^{2}T+K^{*}T_{3}\nabla^{2}\theta .$$

Additionally, as established by Gaurav and Kulkarni [58], in the absence of internal heat generation (Q=0) within the solid, the thermal conductivity of the TPL theory in the setting of Equation (18) is demarcated as follows:

(19) $$T_{1}\left(\rho C_{E}\ddot{\vartheta }+\gamma T_{0}\ddot{e}\right)=\;\left(KT_{2}+K^{*}T_{3}\right)\nabla^{2}T.$$

The more accurate fractional-order three-phase-lag form in Equation (9) is derived via Equation (19), as expressed via the following formula:

(20) $$T_{1}\left(\rho C_{E}\frac{\partial \theta }{\partial t}+\gamma T_{0}z\frac{\partial }{\partial t}\left(\frac{\partial^{2}w}{\partial x^{2}}\right)\right)=\;\left(KT_{2}+K^{*}T_{3}\right)\left(\frac{\partial^{2}\theta }{\partial x^{2}}+\frac{\partial^{2}\theta }{\partial z^{2}}\right),$$

where the fractional operators $T_{1}$, $T_{2}$, $T_{3}$ in Equations (14), (16) and (18)–(20) are given by:

(21) $$T_{1}=1+\frac{\tau_{1}^{\alpha }}{\alpha !}\frac{\partial^{\alpha }}{\partial t^{\alpha }}+\frac{\tau_{1}^{2\alpha }}{2\alpha !}\frac{\partial^{2\alpha }}{\partial t^{2\alpha }},T_{2}=1+\frac{\tau_{2}^{\alpha }}{\alpha !}\frac{\partial^{\alpha }}{\partial t^{\alpha }}+\frac{\tau_{2}^{2\alpha }}{2\alpha !}\frac{\partial^{2\alpha }}{\partial t^{2\alpha }},\;T_{3}=1+\frac{\tau_{3}^{\alpha }}{\alpha !}\frac{\partial^{\alpha }}{\partial t^{\alpha }}+\frac{\tau_{3}^{2\alpha }}{2\alpha !}\frac{\partial^{2\alpha }}{\partial t^{2\alpha }},$$

and α denotes a coefficient fractional order of the time derivative.

## 4. Analytical Solution

To derive an analytical solution, this study posits that the temperature increase is thermally insulating across the thickness and exhibits sinusoidal variation in that direction. The temperature distribution can be mathematically expressed as follows:

(22) $$\theta \left(x,z,t\right)=\theta \left(x,t\right)\sin\left(\frac{\pi z}{h}\right).$$

By substituting this temperature variation (Equation (22)) into the equation of motion (Equation (8)), the governing equation can be expressed as follows:

(23) $$\frac{\partial^{4}w}{\partial x^{4}}-\frac{{e_{0}K}_{1}+K_{2}}{IE+e_{0}K_{2}}\frac{\partial^{2}w}{\partial x^{2}}+\frac{\rho A}{IE+e_{0}K_{2}}\frac{\partial^{2}}{\partial t^{2}}\left(w-e_{0}\frac{\partial^{2}w}{\partial x^{2}}\right)+\frac{K_{1}}{IE+e_{0}K_{2}}w+\frac{\alpha_{T}}{IE+e_{0}K_{2}}\frac{24}{b\pi^{2}}\frac{\partial^{2}\theta }{\partial x^{2}}=0.$$

Applying Equations (7) and (22), the expression for the moment (M) is given by:

(24) $$M\left(x,t\right)=e_{0}\rho A\frac{\partial^{2}w\left(x,t\right)}{\partial t^{2}}+e_{0}K_{1}w\left(x,t\right)-\left(IE+e_{0}K_{2}\right)\frac{\partial^{2}w\left(x,t\right)}{\partial x^{2}}-\frac{{24\alpha }_{T}}{b\pi^{2}}\theta .$$

A generalized heat conduction equation can be formulated using Equations (20) and (22), as follows:

(25) $$T_{1}\left(\rho C_{E}\frac{\partial \theta }{\partial t}+\gamma T_{0}z\frac{\partial }{\partial t}\left(\frac{\partial^{2}w}{\partial x^{2}}\right)\right)=\;\left(KT_{2}+K^{*}T_{3}\right)\left(\frac{\partial }{\partial x^{2}}-\frac{\pi^{2}}{b^{2}}\right)\theta ,\;$$

where the values of the parameters $T_{1},\;T_{2},$ and $T_{3}$ are determined according to Equation (21).

For subsequent investigations, the following dimensionless variables were introduced:

(26) $$\begin{array}{c} \;\left\{x^{\prime },z^{\prime },u^{\prime },w^{\prime },b^{\prime }\right\}=\frac{1}{L}\left\{x^{\prime },z^{\prime },u^{\prime },w^{\prime },b^{\prime }\right\},\;\theta^{\prime }=\frac{\theta }{T_{0}},\;c_{0}=\sqrt{\frac{E}{\rho }},\;e_{0}^{\prime }=\frac{e_{0}}{L^{2}},\\ \left\{t^{\prime },{\tau^{\prime }}_{1},{\tau^{\prime }}_{2},{\tau^{\prime }}_{3}\right\}=\frac{c_{0}}{L}\left\{t^{\prime },{\tau^{\prime }}_{1},{\tau^{\prime }}_{2},{\tau^{\prime }}_{3}\right\},\;\;M^{\prime }=\frac{M}{ALE},\;\;c_{0}L=\frac{K}{\rho C_{E}}=\frac{K^{*}}{\rho C_{E}}. \end{array}$$

By employing these dimensionless relations (Equation (26)), governing Equations (23)–(25) can be simplified as follows:

(27) $$\frac{\partial^{4}w}{\partial x^{4}}-\Upsilon_{1}\frac{\partial^{2}w}{\partial x^{2}}+\Upsilon_{2}\frac{\partial^{2}}{\partial t^{2}}\left(w-e_{0}\frac{\partial^{2}w}{\partial x^{2}}\right)+\Upsilon_{3}w=-\Upsilon_{4}\frac{\partial^{2}\theta }{\partial x^{2}}=0,$$

(28) $$M\left(x,t\right)=e_{0}\frac{\partial^{2}w\left(x,t\right)}{\partial t^{2}}+\Upsilon_{5}w\left(x,t\right)-\Upsilon_{6}\frac{\partial^{2}w\left(x,t\right)}{\partial x^{2}}-\Upsilon_{7}\theta ,$$

(29) $$T_{1}\left(\frac{\partial \theta }{\partial t}+\Upsilon_{8}\frac{\partial }{\partial t}\left(\frac{\partial^{2}w}{\partial x^{2}}\right)\right)=T_{4}\;\left(\frac{\partial^{2}}{\partial x^{2}}-\Upsilon_{9}\right)\theta ,$$

where the parameter $\;T_{1}$ is calculated using Equation (21), $\;T_{4}$ is defined by the fractional differential-operator $T_{4}=2+\frac{\tau_{2}^{\alpha }}{\alpha !}\frac{\partial^{\alpha }}{\partial t^{\alpha }}+\frac{\tau_{2}^{2\alpha }}{2\alpha !}\frac{\partial^{2\alpha }}{\partial t^{2\alpha }}+\frac{\tau_{3}^{\alpha }}{\alpha !}\frac{\partial^{\alpha }}{\partial t^{\alpha }}+\frac{\tau_{3}^{2\alpha }}{2\alpha !}\frac{\partial^{2\alpha }}{\partial t^{2\alpha }},$ and the coefficients $\gamma_{1}$ to $\gamma_{9}$ are defined as follows:

(30) $$\;\begin{array}{c} \Upsilon_{1}=\frac{{e_{0}L^{2}K}_{1}+{L^{2}K}_{2}}{IE+e_{0}K_{2}}\;\;,\;\;\;\;\;\;\;\;\;\;\Upsilon_{2}=\frac{L^{2}\rho Ac_{0}^{2}}{IE+e_{0}K_{2}}\;\;,\;\;\;\;\;\;\;\;\Upsilon_{3}=\frac{L^{4}K_{1}}{IE+e_{0}K_{2}}\;\;,\;\;\;\;\;\;\;\;\;\;\;\;\Upsilon_{4}=\frac{1}{b\pi^{2}}\frac{{24\alpha }_{T}T_{0}}{IE+e_{0}K_{2}}\;,\\ \Upsilon_{5}=\frac{{e_{0}L}^{2}K_{1}}{AE},\;\;\Upsilon_{6}=\frac{{e_{0}L}^{2}K_{2}+IE}{L^{2}AE}\;\;,\;\;\;\;\Upsilon_{7}=\frac{{24\alpha }_{T}T_{0}}{L^{2}AE\pi^{2}}\;\;,\;\;\;\;\;\;\Upsilon_{8}=\frac{\pi^{2}\gamma {Lbc_{0}T}_{0}}{24K}\;\;,\;\;\;\;\Upsilon_{9}={\left(\frac{\pi }{b}\right)}^{2}\;. \end{array}\;$$

In this context, the dimensionless equation of motion is Equation (27), the moment (M) within the small-scale beam is represented by Equation (28), and the dimensionless coupled heat conduction equation is Equation (29).

## 5. Initial and Boundary Conditions and Problem Solution

To address this problem, it is crucial to ascertain initial and boundary constraints. The initial homogeneous constraints are mathematically defined as follows:

(31) $$w\left(x,0\right)=\frac{\partial w(x,0)}{\partial t}\;=\;\Theta \left(x,0\right)=\frac{\partial T(x,0)}{\partial t}=0.$$

We assume that both ends of the small-scale beam are a simply supported boundary, represented by

(32) $$\frac{\partial^{2}w\left(0,t\right)}{\partial x^{2}}=\frac{\partial^{2}w\left(L,t\right)}{\partial x^{2}}=w\left(0,t\right)=w\left(L,t\right)=0.\;$$

Moreover, we consider that the small-scale beam is thermally loaded by slope-type heating, resulting in

(33) $$\theta \left(x,t\right)=T_{0}\{\begin{array}{c} 0\;:\;\;\;\;\;\;\;\;\;\;\;\;\;\;t\leq 0\\ 1\;:\;\;\;\;\;\;\;\;\;\;\;\;\;t>t_{0}\\ \;\;\;\;\frac{t}{t_{0}}\;:\;\;\;\;\;\;\;0\leq t\leq t_{0}. \end{array}\;$$

Here, $t_{0}$ is a slope-type parameter and $T_{0}$ is a constant. Additionally, the temperature at the end boundary must satisfy the following formula:

(34) $$\frac{\partial \theta }{\partial x}=0\;with\;x=L.$$

To solve this problem, we employed the Laplace transform, which is considered by the integral formula for the function L(x,t):

(35) $$\bar{L}\left(x,t\right)=\int_{0}^{\infty }L\left(x,t\right)e^{-st}dt.$$

Applying the Laplace transform to both sides of Equations (27) and (28), and considering the homogeneous initial conditions defined by Equation (31), we derive the following field equations in the Laplace transform domain:

(36) $$\begin{array}{c} \frac{d^{4}\bar{w}}{dx^{4}}-\beta_{1}\frac{d^{2}\bar{w}}{dx^{2}}+\beta_{2}\bar{w}=-\beta_{3}\frac{d^{2}\bar{\theta }}{dx^{2}},\\ \left(\frac{d^{2}}{dx^{2}}-\beta_{4}\right)\bar{\theta }=-\beta_{5}\frac{d^{2}\bar{w}}{dx^{2}},\;\;\;\;\;\;\;\;\;\;\;\;\;\; \end{array}$$

(37) $$\bar{M}\left(x,s\right)=\beta_{6}\bar{w}-\beta_{7}\frac{d^{2}\bar{w}}{dx^{2}}-\beta_{8}\bar{\theta },$$

where the coefficients $\beta_{1}$ to $\beta_{8}$ are defined as:

$$\beta_{1}=\Upsilon_{1}+s^{2}e_{0}\Upsilon_{2},\;\;\beta_{2}=s^{2}\Upsilon_{2}+\Upsilon_{3},\;\;\beta_{3}=\gamma_{4},\;\;\beta_{4}=\delta +\Upsilon_{9},\;\;\;\;\;\;\;\;\;\;\;\;\beta_{5}=\delta \Upsilon_{8},\;$$

$$\;\beta_{6}=e_{0}s^{2}+\gamma_{5}\;,\;\;\;\;\;\;\;\;\;\;\;\;{\;\beta }_{7}=\Upsilon_{6},\;\;\;\;\;\;\;\;\;\;\;\;\;\beta_{8}=\Upsilon_{7},\;\;\;\;\;\;\;\;\;\;\delta =\frac{s+\frac{s^{\alpha +1}}{\alpha !}\tau_{1}^{\alpha }+\frac{s^{2\alpha +1}}{2\alpha !}\tau_{1}^{2\alpha }}{2s+\frac{s^{\alpha +1}}{\alpha !}\tau_{2}^{\alpha }+\frac{s^{2\alpha +1}}{2\alpha !}\tau_{2}^{2\alpha }+\frac{s^{\alpha +1}}{\alpha !}\tau_{3}^{\alpha }+\frac{s^{2\alpha +1}}{2\alpha !}\tau_{3}^{2\alpha }}.\;$$

By eliminating either $\bar{\theta }$ or $\bar{w}$ from Equation (32), the following formula is obtained:

(38) $$\left(D^{6}-A_{1}D^{4}+A_{2}D^{2}-A_{3}\right)\left\{\bar{w},\bar{\theta }\right\}\left(x\right)=0,$$

where, in this equation, $A_{1}$, $A_{2},\;A_{3}$ and D are given by

(39) $$A_{1}=\beta_{3}\beta_{5}+\beta_{1}+\beta_{4},\;\;\;\;\;\;\;A_{2}=\beta_{2}+\beta_{1}\beta_{4},\;\;\;\;\;\;\;A_{3}=\beta_{2}\beta_{4},\;\;\;\;\;\;\;D=\frac{d}{dx}.$$

Equation (38) can be further analyzed and expressed as:

(40) $$\left(D^{2}-m_{1}^{2}\right)\left(D^{2}-m_{2}^{2}\right)\left(D^{2}-m_{3}^{2}\right)\left\{\bar{w},\bar{\theta }\right\}\left(x\right)=0,$$

in which $m_{i}^{2}$ and i=1,2,3 are the roots of

(41) $$m^{6}-Am^{4}+Bm^{2}-C=0.$$

The accuracy of Equation (41) in the Laplace transform domain can be expressed as follows:

(42) $$\left\{\bar{w},\bar{\theta }\right\}\left(x\right)=\sum_{i=1}^{3}\left({\left\{1,\Omega_{i}\right\}\beta }_{i}e^{-m_{i}x}+{\left\{1,\Omega_{i+3}\right\}\beta }_{i+3}e^{m_{i}x}\right).$$

By integrating Equation (42) with Equation (36), we obtain

(43) $$\Omega_{i}=-\frac{m_{i}^{2}\beta_{5}}{m_{i}^{2}-\beta_{4}}.$$

According to Equation (42), the displacement can be written as:

(44) $$\bar{u}\left(x\right)=-z\frac{d\bar{w}}{dx}=z\sum_{i=1}^{3}m_{i}\left(\beta_{i}e^{-m_{i}x}-\beta_{i+3}e^{m_{i}x}\right).$$

By substituting Equation (42) into Equation (37), we determine the solution for moment $\bar{M}$, which is characterized as

(45) $$\bar{M}\left(x\right)=-\sum_{i=1}^{3}\left(m_{i}^{2}\beta_{6}{+\beta_{7}\Omega }_{i}\right)\left(\beta_{i}e^{-m_{i}x}-\beta_{i+3}e^{m_{i}x}\right).$$

Additionally, the strain was defined as

(46) $$\bar{e}\left(x\right)=\frac{d\bar{u}}{dx}=-z\sum_{i=1}^{3}m_{i}^{2}\left(\beta_{i}e^{-m_{i}x}-\beta_{i+3}e^{m_{i}x}\right).$$

The Laplace transform adjusts boundary conditions (29)–(31) to the following order:

(47) $$\begin{array}{c} \bar{w}\left(0,s\right)=\bar{w}\left(L,s\right)=0,\;\;\frac{\partial^{2}\bar{w}(0,s)}{\partial x^{2}}=\frac{\partial^{2}\bar{w}(L,s)}{\partial x^{2}}=0\\ \frac{\partial \bar{\theta }\left(0,s\right)}{\partial x}=T_{0}\left(\frac{1-e^{-st_{0}}}{{s^{2}t}_{0}}\right),\;\;\;\;\;\;\;\;\;\;\;\frac{\partial \bar{\theta }\left(L,s\right)}{\partial x}=0. \end{array}$$

By incorporating Equation (42) into these boundary conditions, a set of six linear equations is determined, denoted as

(48) $$\begin{array}{cc} \sum_{i=1}^{3}\left(\beta_{i}+\beta_{i+1}\right)=0,\;\;\;\;\;\;\;\;\;\;\; & \sum_{i=1}^{3}\left(\beta_{i}e^{-m_{i}L}+\beta_{i+1}e^{m_{i}L}\right)=0 \end{array}.$$

(49) $$\begin{array}{cc} \sum_{i=1}^{3}m_{i}^{2}\left(\beta_{i}+\beta_{i+1}\right)=0,\;\;\;\;\;\;\;\;\;\;\;\;\; & \sum_{i=1}^{3}m_{i}^{2}\left(\beta_{i}e^{-m_{i}L}+\beta_{i+1}e^{m_{i}L}\right)=0 \end{array},$$

(50) $$\begin{array}{cc} \sum_{i=1}^{3}m_{i}\left({\Omega_{i}\beta }_{i}-{\Omega_{i+1}\beta }_{i+1}\right)=-T_{0}\left(\frac{1-e^{-st_{0}}}{{s^{2}t}_{0}}\right),\;\; & \sum_{i=1}^{3}m_{i}\left({\Omega_{i}\beta }_{i}e^{-m_{i}L}-{\Omega_{i+1}\beta }_{i+1}e^{m_{i}L}\right)=0. \end{array}$$

The set of linear equations presented in Equations (48)–(50) was solved to determine the unknown parameters $\;\beta_{i}$, (i=1, 2,…,6), which determine the vibrational and thermal response of the small-scale beam. Numerical results relevant to physical field studies were obtained via the Riemann sum approximation technique or mathematical Laplace-inversion technique [17,59]. This system is an essential step in finding an analytical solution to the coupled thermoelasticity problem, as it ensures the stability and accuracy of the temperature (Θ), displacement (u), moment (M) and deflection (w). By approximating the inversion integral as a finite sum of exponential and trigonometric limits, the robustness of the model is confirmed, ensuring that the resulting wave propagation characteristics remain physically consistent with the fractional three-phase-lag (FTPL) model.

## 6. Special Cases

The fractional three-phase-lag heat conduction equation derived from the two-temperature theory (Equation (18)) can be simplified to align with several recognized thermoelasticity models under specific conditions.

Case 1: If the thermodynamic temperature is the conductive temperature (T=ϑ) and ($\tau_{1}=\tau_{2}=0$) with the coupling parameter ($K^{*}=0$), then Equation (18) represents the fractional classical coupled thermoelasticity (FCTE) model.
Case 2: When the conductive temperature is the thermodynamic temperature (T=ϑ) with $(\tau_{1}>0$) and ($\tau_{2}=K^{*}=0$), Equation (18) leads to the fractional Lord and Shulman (FLS) model.
Case 3: When the coupling parameter $K^{*}\neq 0$ and the conductive temperature is the thermodynamic temperature (T=ϑ) with ($\tau_{1}>0,\tau_{2}>0$), Equation (18) signifies the fractional three-phase-lag (FTPL) model.

## 7. Numerical Results

This section examines the influence of different models of thermoelasticity, a two-parameter elastic foundation, and fractional-order parameters on the distributions of temperature, displacement, deflection, and moment. The specific material properties utilized for this analysis, as provided by Adam and Abouelregal [17] for a micro-scale-beam, are shown in Table 1 below:

In addition, the analysis was grounded in the theoretical framework described in the previous sections. Our results, depicted in Figure 2, Figure 3, Figure 4, Figure 5, Figure 6, Figure 7, Figure 8, Figure 9, Figure 10, Figure 11, Figure 12 and Figure 13 and Table 2, Table 3, Table 4, Table 5, Table 6, Table 7, Table 8, Table 9, Table 10, Table 11, Table 12 and Table 13, in conjunction with the results of other studies, affirm the originality of our study.

### 7.1. Analysis of the Effects of Different Models of Thermoelasticity

**Figure 2:**
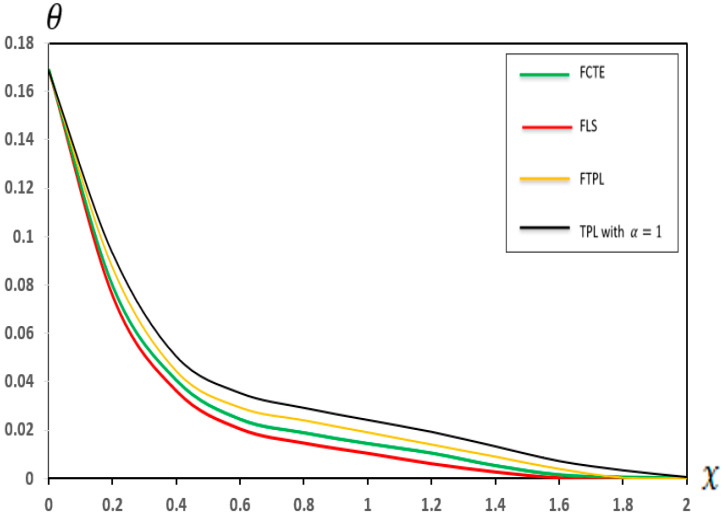
Temperature (θ) in different models of thermoelasticity.

**Figure 3:**
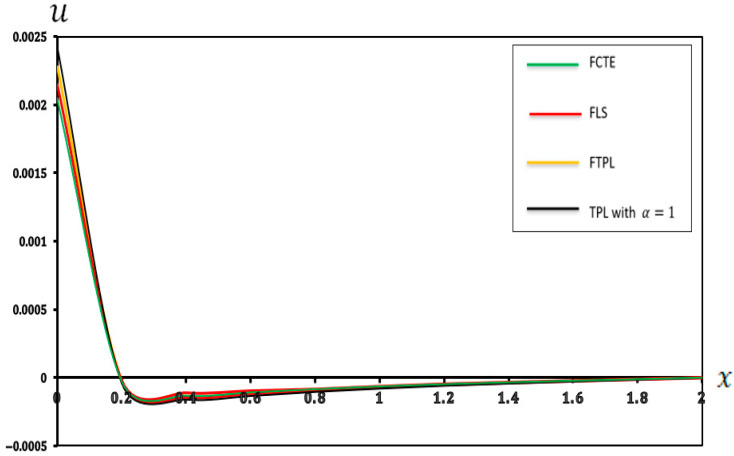
Displacement (u) in different models of thermoelasticity.

**Figure 4:**
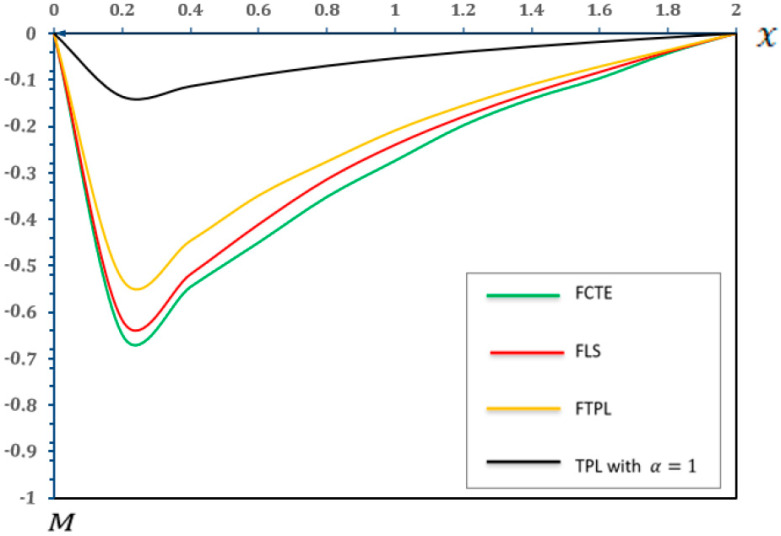
Moment (M) in different models of thermoelasticity.

**Figure 5:**
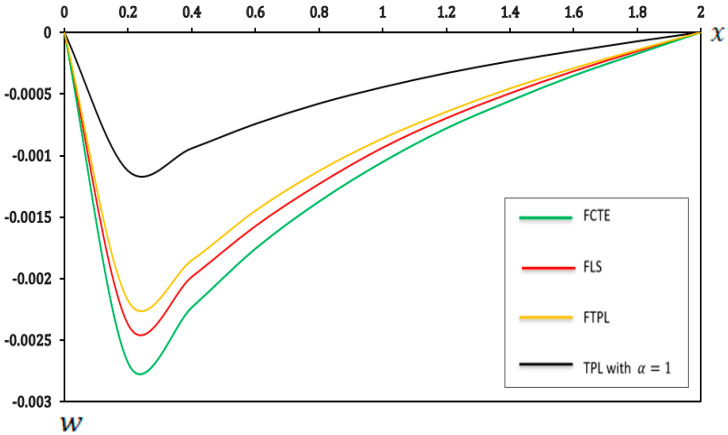
Deflection (w) in different models of thermoelasticity.

In this section, we present a graphical demonstration of the distributions of temperature (Θ), displacement (u), moment (M) and deflection (w) for the different thermoelastic models (FCTE, FLS, FTPL, and TPL with α=1) with a fractional-order parameter (α=0.4), nonlocal parameter $e_{0}=0.2$, two-parameter elastic foundation $\left(K_{1}=0.1,\;K_{2}=0.05\right)$ and values $\left(\tau_{1}=0.8,\tau_{2}=0.4,\;and\;\tau_{3}=0.2\right),$ and other parameters, such as those specified in Section 7. Figure 2, Figure 3, Figure 4 and Figure 5 and Table 2, Table 3, Table 4 and Table 5 present the results of the application of thermoelastic and vibration analysis to the modeling and analysis of a microbeam subjected to thermoelastic influences, based on a two-parameter elastic foundation. These results illustrate the differences and similarities between thermoelastic models, which are gaining importance in various engineering fields, especially in micro-electromechanical systems (MEMSs) and sensors. Additional explanations from Figure 2, Figure 3, Figure 4 and Figure 5 and Table 2, Table 3, Table 4 and Table 5 can be summarized as follows:

**Table 2:** Effects of different models of thermoelasticity on temperature (θ).

X	FCTE	FLS	FTPL	TPL with α=1
0	0.168506	0.168506	0.168506	0.168506
0.2	0.0752087	0.07924313	0.0872754	0.09314605
0.4	0.03603204	0.04031544	0.04448751	0.05038328
0.6	0.02036032	0.02424035	0.0294679	0.03525406
0.8	0.01452036	0.01865242	0.02418007	0.02910328
1	0.01032402	0.01420725	0.01918624	0.02410086
1.2	0.00602724	0.01027654	0.01417208	0.01917102
1.4	0.00261327	0.00510274	0.00917905	0.01317039
1.6	0.00023516	0.00132102	0.00412008	0.00710322
1.8	0.00014385	0.000327042	0.00048709	0.00328027
2	0.000047309	0.000162548	0.000290403	0.000490124

Figure 2 and Table 2 demonstrate the variation in the temperature distribution along the x-axis of the micro-scale beam for different flexible thermal models (FCTE, FLS, FTPL and TPL with α=1). In all three models, the temperature (θ) reached its maximum at (X=0). Subsequently, the temperature (θ) decreases as the distance (X) increases, which aligns with the boundary conditions. Moreover, the analysis revealed that the selection of a flexible thermal model significantly influences the predicted temperature distribution within the beam. In particular, the FTPL model forecasts a distinct temperature distribution that incorporates time-lag effects. Comparing this model with simpler alternatives, such as (FCTE, FLS, or TPL with α=1), enables engineers to evaluate the significance of these time-lag terms in accurately predicting thermal behavior at the micro-scale level. Furthermore, Figure 2 shows the key data related to microsensors and microthermal actuators, where the temperature distribution within a micro-scale beam is a critical factor affecting its design and performance. Previous research [59] has consistently revealed a similar trend in the temperature distribution within beams, with the temperature peaking at (X = 0) and then decreasing with increasing distance. The present results, as shown in Figure 2 and Table 2, are consistent with this pattern across all the thermoelastic models (FCTE, FLS, FTPL, and TPL). However, the temperature distribution shown in Figure 2 and Table 2 represents critical data for the design and performance validation of microsensors and microthermal actuators, where the thermal field is a primary operational factor. This study’s detailed comparative analysis elucidates the unique impact of the time-lag properties inherent in the (FTPL) model, offering enhanced predictive accuracy in thermal characterization beyond what standard thermoelastic models provide.

**Table 3:** Effects of different models of thermoelasticity on displacement (u).

X	FCTE	FLS	FTPL	TPL with α=1
0	0.002278	0.002146	0.002437	0.002047
0.2	−0.000028	−0.000023	−0.000035	−0.000025
0.4	−0.000142	−0.000113	−0.000159	−0.000132
0.6	−0.000116	−0.000098	−0.000132	−0.000108
0.8	−0.000093	−0.000085	−0.000103	−0.000085
1	−0.000070	−0.000064	−0.000079	−0.000064
1.2	−0.000052	−0.000047	−0.000058	−0.000048
1.4	−0.000037	−0.000034	−0.000042	−0.000034
1.6	−0.000024	−0.000022	−0.000027	−0.000022
1.8	−0.000012	−0.000011	−0.000013	−0.000011
2	0.000000	0.000000	0.000000	0.000000

Figure 3 shows the displacement (u) of the points along the microbeam length under thermoelastic loading conditions. Notably, all the models (FCTE, FLS, FTPL, and TPL with α=1) have negative displacement values within the interval (0.2 < x < 1.4), eventually converging to zero. In addition, from Figure 3 and Table 3, the incidence of both positive and negative displacements indicates complex internal stresses and deformations resulting from the combined effects of thermal and mechanical loads. Compared with simpler theories, the (FTPL) model can predict displacement owing to its more comprehensive approach to the material response to thermal and mechanical stimuli. This understanding is essential for engineers to analyze the axial extension or contraction of a beam under specified conditions. Precise quantification of this complex axial-strain field is fundamentally important for the design and operational lifespan of high-precision micro-actuators and MEMS resonators. Engineers must use models like the (FTPL) to accurately determine the location and magnitude of peak tensile and compressive strains to prevent material fatigue and set critical strain tolerance limits for the component under thermal cycling. The model ensures reliable analysis of the micro-scale beam’s extension or contraction, a key parameter in ensuring mechanical reliability.

**Table 4:** Effects of different models of thermoelasticity on moment (M).

X	FCTE	FLS	FTPL	TPL with α=1
0	−0.000425	−0.000425	−0.000425	−0.000425
0.2	−0.648203	−0.617602	−0.529802	−0.136497
0.4	−0.545301	−0.518482	−0.446411	−0.114151
0.6	−0.449704	−0.412081	−0.348903	−0.090503
0.8	−0.351372	−0.314365	−0.276207	−0.070166
1	−0.273790	−0.239861	−0.208901	−0.053779
1.2	−0.197680	−0.179238	−0.155461	−0.040121
1.4	−0.140210	−0.127011	−0.112506	−0.028409
1.6	−0.096543	−0.083247	−0.071473	−0.018218
1.8	−0.042859	−0.039723	−0.036204	−0.008870
2	0.000000	0.000000	0.000000	0.000000

According to Figure 4 and Table 4, the FTPL model predicts lower magnitudes of internal moment (M) than the FCTE and FLS models. From a physical interpretation standpoint, the FTPL model’s prediction of lower moments indicates that the material, when described by this fractional-order theory, displays more compliant or stress-redistributing behavior under thermoelastic loading. This behavior could be attributed to the inherent nonlocal and memory effects captured by fractional calculus, which allow for more distributed energy dissipation or load sharing within the microstructure. This observation aligns with the findings of study [59]. A study [59] also observed that generalized thermoelastic models, particularly those incorporating phase-lag terms, tend to predict reduced mechanical responses, such as moments, under similar thermoelastic loading conditions in beams. This consistency reinforces the validity of the FTPL model predictions in capturing the nuanced thermoelastic behavior of such microstructures. The magnitude of the maximum bending moment is a critical design criterion directly related to the structural integrity and fatigue life of micro-electromechanical systems (MEMSs). The (FTPL) model provides an accurate prediction of this maximum stress, indicating that the inclusion of thermal and nonlocal relaxation effects suggests the component experiences less internal mechanical stress than predicted by simpler fractional models (FCTE and FLS). The use of this advanced model is crucial for accurately determining the maximum operating thermal load and ensuring the long-term reliability of micro-actuators and resonators operating under high thermomechanical cycles.

**Table 5:** Effects of different models of thermoelasticity on deflection (w).

X	FCTE	FLS	FTPL	TPL with α=1
0	0.000000	0.000000	0.000000	0.000000
0.2	−0.002369	−0.001124	−0.002687	−0.002179
0.4	−0.001984	−0.000941	−0.002237	−0.001852
0.6	−0.001572	−0.000743	−0.001760	−0.001450
0.8	−0.001231	−0.000578	−0.001376	−0.001126
1	−0.000936	−0.000445	−0.001055	−0.000863
1.2	−0.000697	−0.000331	−0.000781	−0.000646
1.4	−0.000495	−0.000235	−0.000558	−0.000456
1.6	−0.000316	−0.000152	−0.000353	−0.000292
1.8	−0.000154	−0.000073	−0.000174	−0.000143
2	0.000000	0.000000	0.000000	0.000000

Table 5 and Figure 5 illustrate that the FTPL model predicts greater deflection of the beam than the specific TPL model with α=1 but less deflection than the FCTE and FSL models. This observation suggests that the incorporation of fractional-order derivatives and three-phase-lag impacts within the FTPL model introduces mechanisms that mitigate the overall deformation of the micro-scale beam in response to the applied stimuli. These mechanisms involve an increase in effective stiffness, as captured by the more complex FTPL model. Additionally, when microstructures such as micro-scale beams are designed, the predicted deflection under thermal or mechanical loads is a critical design parameter. Compared to other theories, the (FTPL) model in Figure 5 and Table 5 provides different estimates of this deflection. Therefore, using the (FTPL) model during the design phase allows engineers to determine more accurate and precise deflection tolerances for thermal and mechanical-loading scenarios. This ensures that the final design aligns with the actual dynamic stability and operational performance of the microdevice, reducing the risk of functional failure or structural collapse predicted by less sophisticated models. Estimating the unique (FTPL) deflection model, derived from its improved representation of thermal and mechanical energy transfer, provides better predictive reliability compared to theories that ignore the phenomena of memory and relaxation.

### 7.2. Analysis of the Effects of a Two-Parameter Elastic Foundation

**Figure 6:**
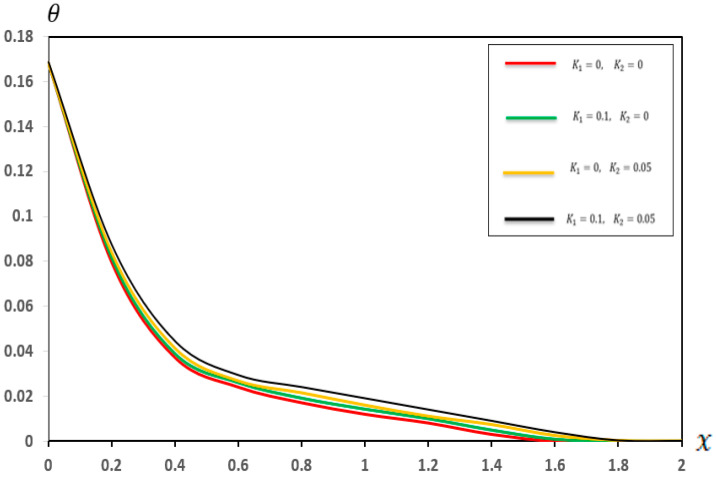
Temperature (θ) in a two-parameter elastic foundation.

**Figure 7:**
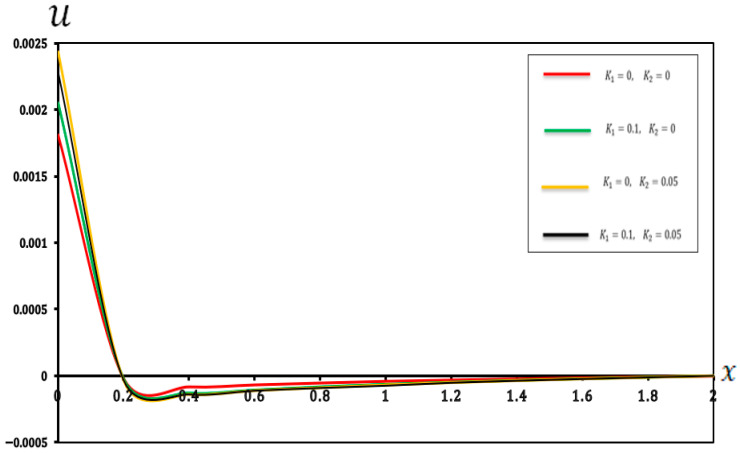
Displacement (u) in a two-parameter elastic foundation.

**Figure 8:**
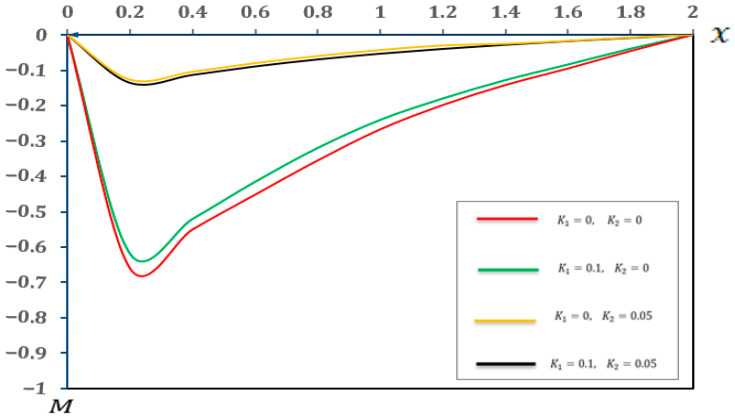
Moment (M) in a two-parameter elastic foundation.

**Figure 9:**
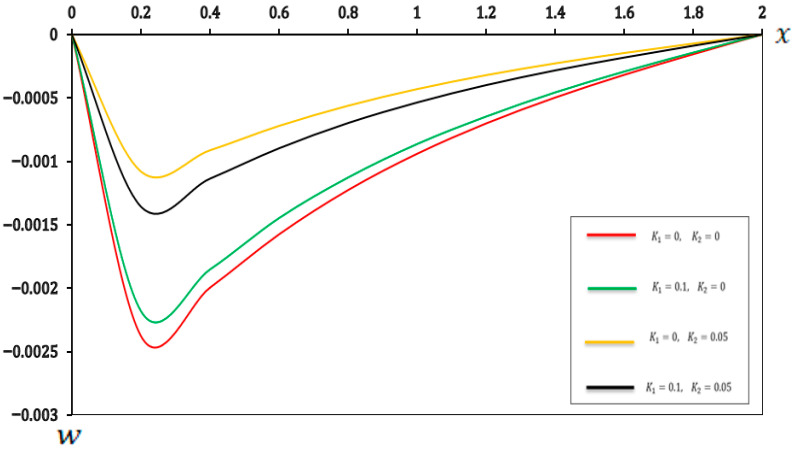
Deflection (w) in a two-parameter elastic foundation model.

In this section, we present a graphical of the distributions of several physical fields for various two-parameter elastic foundation models ($\left(K_{1}=0.1,{\;K}_{2}=0.05\right)$, $\left(K_{1}=0.1,{\;K}_{2}=0\right)$, $\left(K_{1}=0,{\;K}_{2}=0.05\right)$, and $\left(K_{1}=0,{\;K}_{2}=0\right)$). The consequences are exposed in Figure 6, Figure 7, Figure 8 and Figure 9 and Table 6, Table 7, Table 8 and Table 9. Furthermore, the results were compared with those in the literature [60] and demonstrated strong agreement with the findings presented in the literature [60]. The main observations from Figure 6, Figure 7, Figure 8 and Figure 9 and Table 6, Table 7, Table 8 and Table 9 can be summarized as follows:

- As shown in Figure 6 and Table 6, the temperature distribution along the beam implies remarkable uniformity, even when the values of ($K_{1}$ and $K_{2}$) are varied. The close alignment of the curves for different ($K_{1}$ and $K_{2})$ values indicates that the thermal behaviour of the micro-scale beam is primarily governed by the applied thermal load and the intrinsic thermal properties of the material. Conversely, the stiffness and shear reactions attributed to the two-parameter elastic foundation exerted a negligible direct influence on the temperature distribution within the microbeam. This finding is highly significant for the modular design of MEMS and NEMS devices. It demonstrates that thermal design and mechanical support design can be treated as largely independent processes. Engineers can optimize the ($K_{1}$) and ($K_{2})$ parameters of the substrate to achieve desired mechanical properties, without significantly altering the expected operating temperature pattern of the beam.
- Based on Figure 7 and Table 7, the displacement (u) was slightly affected by the foundation coefficients, especially close to the ends of the beam. The increasing values of the foundation coefficients corresponded to a slight decrease in displacement, indicating that the parameter ($K_{1}=0.1$) and the reaction parameter ($K_{2}=0.05$) provided better resistance to the general deformation of the microbeam. This resistance slightly limited the axial movement, resulting in a significant reduction in displacement. These results provide a quantitative basis for selecting material properties and intermediate layer thicknesses that mimic a two-parameter foundation. By matching the theoretical values of ($K_{1}$) and ($K_{2})$ with the properties of the polymer material or the compatible layer beneath the micro-scale beam, engineers can structurally customize the energy-dissipation path, minimize mechanical to thermal energy transfer and maximize dynamic stability.
- Figure 8 and Table 8 explain the significant impact of the two-parameter elastic foundation on the moment (M) of the beams. Our results suggest that higher values of the parameter elastic foundation consistently resulted in lower moments along the beam. The varying distribution of moments with the foundation stiffness confirms that a stiffer foundation provides greater support to the microbeam, reducing its tendency to bend under numerous applied loads. Moreover, the parameter $(K_{2}$) played a crucial role in distributing the load more efficiently, which in turn reduced the moment required for equilibrium. This is notably true for the design and operation of micro-electromechanical systems (MEMSs) and other microstructures supported by flexible substrates, where supervision of internal stresses is essential for prolonging device life and functionality. For micro-actuators relying on precise thermal deformation, the foundation’s stiffness must be factored in to prevent unintended mechanical coupling. These results allow for the accurate calibration of the thermal load required to achieve a specific deflection, as the reduced moment means less energy is wasted on internal stress and more is channeled into desired actuation.
- Similarly, Figure 9 and Table 9 show that the deflection  (w) of the small-scale beam is significantly affected by the two-parameter elastic foundation. We observed that higher values of ($K_{1}$ and $K_{2})$ resulted in a significantly reduced deflection. The stiffer $\left(K_{1}=0.1\right)$ parameter directly resists beam deflection. This effect is enhanced by the $\left(K_{2}=0.05\right)$ parameter, which provides additional support and helps distribute the load, further reducing deflection. This feature is critical for the structural design of micro-electromechanical systems (MEMSs) and other microstructures supported by flexible substrates, where precise deformation control is crucial for maintaining active accuracy and reliability. The high sensitivity of the beam deflection (w) pattern to the base parameters allows for the development of finely tuned mechanical filters. By incorporating electrostatically controlled layers capable of simultaneously modifying the effective values of ($K_{1}$) and ($K_{2})$, the beam stiffness can be precisely altered, enabling frequency tuning without the need for external mechanical adjustments.

**Table 6:** Effects of a two-parameter elastic foundation on temperature (θ).

X	$K_{1}=0,$ $K_{2}=0$	$K_{1}=0.1,$ $K_{2}=0$	$K_{1}=0,$ $K_{2}=0.05$	$K_{1}=0.1,$ $K_{2}=0.05$
0	0.168506	0.168506	0.168506	0.168506
0.2	0.07980763	0.08176325	0.08420037	0.0872754
0.4	0.03736042	0.03904241	0.04141028	0.04448751
0.6	0.02416127	0.02612782	0.02682046	0.0294679
0.8	0.01721225	0.01922518	0.02151875	0.02418007
1	0.01209317	0.01431702	0.01602186	0.01918624
1.2	0.00821012	0.01012109	0.01110917	0.01417208
1.4	0.00314016	0.00501678	0.00740101	0.00917905
1.6	0.00010628	0.00092809	0.00250982	0.00412008
1.8	0.00002865	0.000128029	0.000280218	0.00048709
2	0.000052175	0.000072084	0.000098425	0.000290403

**Table 7:** Effects of a two-parameter elastic foundation on displacement (u).

X	$K_{1}=0,$ $K_{2}=0$	$K_{1}=0.1,$ $K_{2}=0$	$K_{1}=0,$ $K_{2}=0.05$	$K_{1}=0.1,$ $K_{2}=0.05$
0	0.001811	0.002047	0.002432	0.002297
0.2	−0.000019	−0.000024	−0.000037	−0.000028
0.4	−0.000081	−0.000130	−0.000139	−0.000140
0.6	−0.000066	−0.000107	−0.000111	−0.000112
0.8	−0.000052	−0.000084	−0.000089	−0.000091
1	−0.000039	−0.000064	−0.000068	−0.000074
1.2	−0.000030	−0.000048	−0.000051	−0.000052
1.4	−0.000021	−0.000034	−0.000036	−0.000037
1.6	−0.000013	−0.000022	−0.000023	−0.000024
1.8	−0.000006	−0.000011	−0.000011	−0.000012
2	0.000000	0.000000	0.000000	0.000000

**Table 8:** Effects of a two-parameter elastic foundation on moment (M).

X	$K_{1}=0,$ $K_{2}=0$	$K_{1}=0.1,$ $K_{2}=0$	$K_{1}=0,$ $K_{2}=0.05$	$K_{1}=0.1,$ $K_{2}=0.05$
0	−0.000425	−0.000425	−0.000425	−0.000425
0.2	−0.619138	−0.658453	−0.136576	−0.126757
0.4	−0.521587	−0.549564	−0.114205	−0.104523
0.6	−0.416281	−0.451342	−0.090035	−0.080219
0.8	−0.320453	−0.354706	−0.070161	−0.060120
1	−0.240896	−0.266892	−0.053761	−0.043077
1.2	−0.180362	−0.198124	−0.040230	−0.030143
1.4	−0.128241	−0.141854	−0.028435	−0.025468
1.6	−0.084208	−0.095132	−0.018219	−0.017253
1.8	−0.039376	−0.045853	−0.008879	−0.007884
2	0.000000	0.000000	0.000000	0.000000

**Table 9:** Effects of a two-parameter elastic foundation on deflection (w).

X	$K_{1}=0,$ $K_{2}=0$	$K_{1}=0.1,$ $K_{2}=0$	$K_{1}=0,$ $K_{2}=0.05$	$K_{1}=0.1,$ $K_{2}=0.05$
0	0.000000	0.000000	0.000000	0.000000
0.2	−0.002375	−0.002178	−0.001083	−0.001354
0.4	−0.001994	−0.001850	−0.000915	−0.001134
0.6	−0.001575	−0.001446	−0.000722	−0.000895
0.8	−0.001226	−0.001126	−0.000563	−0.000697
1	−0.000940	−0.000863	−0.000432	−0.000535
1.2	−0.000701	−0.000646	−0.000321	−0.000398
1.4	−0.000497	−0.000456	−0.000228	−0.000282
1.6	−0.000318	−0.000292	−0.000146	−0.000181
1.8	−0.000155	−0.000143	−0.000071	−0.000088
2	0.000000	0.000000	0.000000	0.000000

### 7.3. Analysis of the Effect of Fractional-Order Parameters

**Figure 10:**
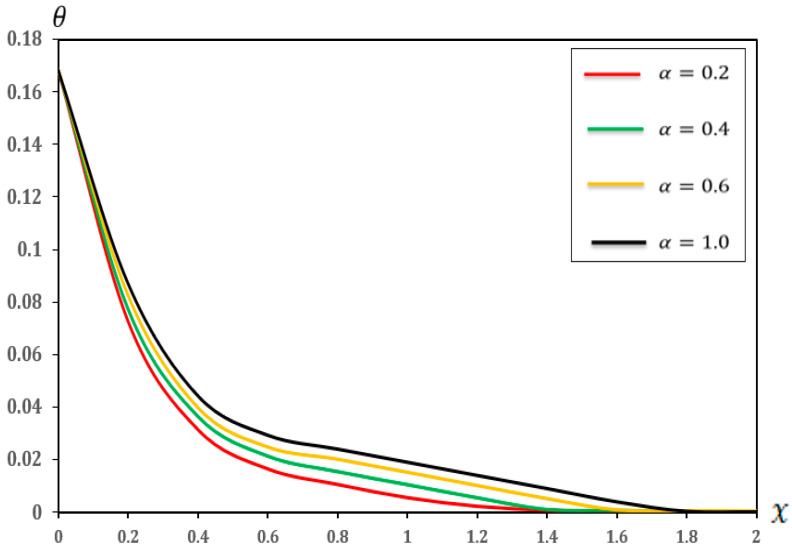
Temperature (θ) with respect to fractional-order parameters.

**Figure 11:**
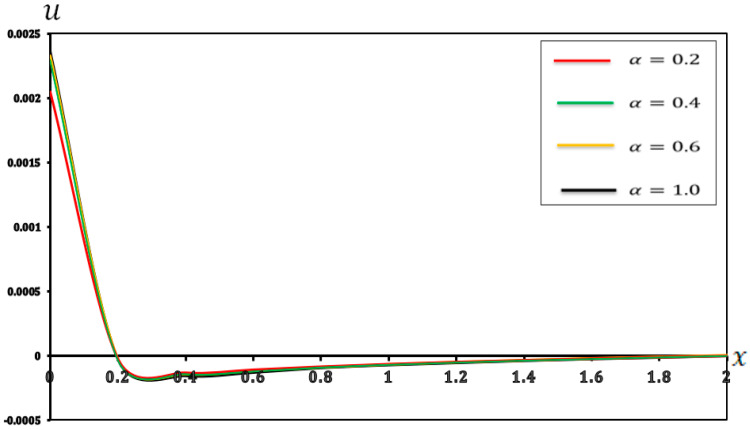
Displacement (u) with respect to fractional-order parameters.

**Figure 12:**
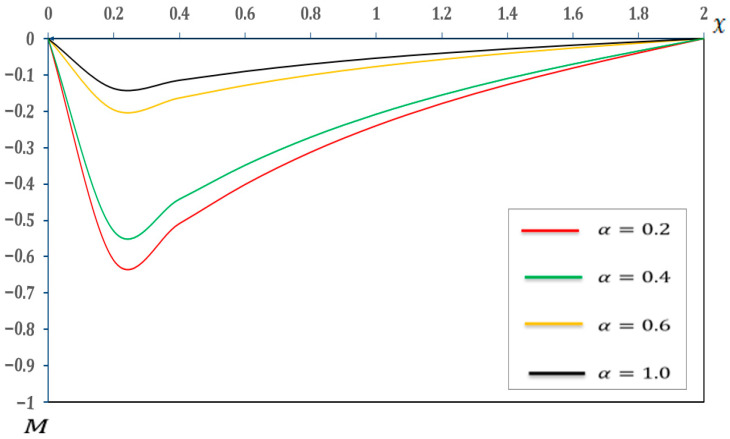
Moment (M) with respect to fractional-order parameters.

**Figure 13:**
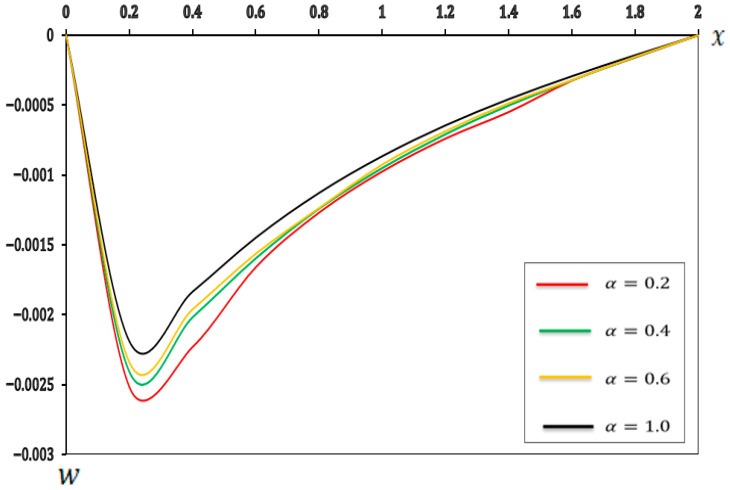
Deflection (w) with respect to fractional-order parameters.

In this section, we examine the impact of fractional-order parameters (α=0.2, α=0.4, α=0.6 and α=1.0)  on temperature (θ), displacement (u), moment (M), and deflection (w) when $\left(K_{1}=0.1,{\;K}_{2}=0.05\right),$ nonlocal parameter $e_{0}=0.2\;$ and values $\tau_{1}=0.8,{\;\tau }_{2}=0.4,\;\mathrm{and}\tau_{3}=0.2$ and other parameters are consistent with those in Section 7. The results are shown in Figure 10, Figure 11, Figure 12 and Figure 13 and detailed in Table 10, Table 11, Table 12 and Table 13.

As proved in Figure 10 and Table 10, as the fractional-order parameter increases from (0.2 to 1.0), the temperature (θ) decreases along the micro-scale beam. The curves suggest that at greater (α) values, the temperature decreased more rapidly than its maximum value at (X = 0), which is consistent with the findings of the study [42]. Additionally, the decrease in temperature with increasing (α) suggests that a greater fractional-order parameter leads to more efficient heat dissipation or distribution within the beam under specific boundary conditions.

As shown in Figure 11 and Table 11, a comparison of the displacement at the left end (X=0) reveals that greater fractional-order parameter (α) values lead to slightly larger positive displacements. However, this trend reverses in the central region of the microbeam, where higher (α) values result in negative displacement. This observation aligns with the findings from other studies, specifically those by [58], underscoring a consistent phenomenon. The intersection of the curves for varying (α) values highlights the non-uniform impact of the fractional-order parameter (α) along the beam. This nonmonotonic impact of (α) implies a complex interplay between the thermal and mechanical fields. Thus, different (α) values can lead to varying expansion or contraction behaviors in distinct regions of the microbeam under applied conditions. This understanding is critical for applications demanding precise motion control, such as advanced precision positioning systems.

The observations from Figure 12 and Table 12 suggest that increasing the fractional-order parameter (α) leads to a decrease in the magnitude of the negative moment (M) along the beam. This suggests that higher fractional-order parameters typically result in lower internal moments. A reduced moment at higher (α) values suggests that the microbeam offers less resistance to bending or that the applied loads and thermal gradients induce lower internal stress. This phenomenon is directly linked to alterations in the effective stiffness properties of the beam, which were predicted by the fractional-order parameter (α). In the realm of structural design, a reduction in the moment (M) can contribute to the creation of lighter and more efficient structures.

Figure 13 and Table 13 illustrate that as the fractional-order parameter (α) increases from (0.2 to 1.0), the magnitude of the negative transverse deflection (w) decreases along the length of the beam. This implies that a higher fractional-order parameter (α) leads to a stiffer bending response from the microbeam, which is consistent with previous studies [40,59]. In essence, a lower deflection coupled with a higher (α) value indicates a greater resistance of the microbeam to bending under specific loads. This makes (α) a critical parameter in micro-scale beam design, directly influencing structural integrity and functional performance.

**Table 10:** Effects of fractional-order parameters on temperature (θ).

X	α=0.2	α=0.4	α=0.6	α=1.00
0	0.168506	0.168506	0.168506	0.168506
0.2	0.072774	0.07743256	0.0827571	0.0872754
0.4	0.0314485	0.0364743	0.03994476	0.04448751
0.6	0.0164231	0.0213208	0.02490047	0.0294679
0.8	0.0104802	0.0154045	0.02024762	0.02418007
1	0.0054325	0.0104348	0.01527653	0.01918624
1.2	0.0021582	0.0054374	0.01024057	0.01417208
1.4	0.0006537	0.0009625	0.00524734	0.00917905
1.6	0.0002789	0.0006508	0.00094522	0.00412008
1.8	0.0000492	0.0002482	0.00064409	0.00048709
2	0.0000279	0.0000491	0.00045474	0.000290403

**Table 11:** Effects of fractional-order parameters on displacement (u).

X	α=0.2	α=0.4	α=0.6	α=1.00
0	0.002363	0.002345	0.002048	0.002297
0.2	−0.000029	−0.000029	−0.000025	−0.000028
0.4	−0.000155	−0.000146	−0.000131	−0.000141
0.6	−0.000129	−0.000121	−0.000108	−0.000120
0.8	−0.000093	−0.000093	−0.000084	−0.000090
1	−0.000071	−0.000071	−0.000065	−0.000069
1.2	−0.000054	−0.000053	−0.000048	−0.000051
1.4	−0.000038	−0.000038	−0.000034	−0.000036
1.6	−0.000024	−0.000024	−0.000022	−0.000024
1.8	−0.000013	−0.000012	−0.000011	−0.000011
2	0.000000	0.000000	0.000000	0.000000

**Table 12:** Effects of fractional-order parameters on moment (M).

X	α=0.2	α=0.4	α=0.6	α=1.00
0	−0.000425	−0.000425	−0.000425	−0.000425
0.2	−0.197125	−0.137056	−0.609504	−0.529605
0.4	−0.164308	−0.114407	−0.508506	−0.442083
0.6	−0.129608	−0.090103	−0.401208	−0.348705
0.8	−0.100976	−0.070205	−0.312561	−0.271607
1	−0.077409	−0.053808	−0.239651	−0.208287
1.2	−0.057811	−0.040250	−0.178681	−0.155413
1.4	−0.040927	−0.028504	−0.126677	−0.110078
1.6	−0.026206	−0.018225	−0.081129	−0.070455
1.8	−0.012807	−0.008978	−0.039605	−0.034370
2	0.000000	0.000000	0.000000	0.000000

**Table 13:** Effects of fractional-order parameters on deflection (w).

X	α=0.2	α=0.4	α=0.6	α=1.00
0	0.000000	0.000000	0.000000	0.000000
0.2	−0.002521	−0.002412	−0.002190	−0.002341
0.4	−0.002233	−0.002021	−0.001834	−0.001961
0.6	−0.001662	−0.001598	−0.001448	−0.001563
0.8	−0.001271	−0.001242	−0.001128	−0.001237
1	−0.000976	−0.000952	−0.000865	−0.000925
1.2	−0.000740	−0.000709	−0.000645	−0.000687
1.4	−0.000550	−0.000503	−0.000457	−0.000487
1.6	−0.000325	−0.000323	−0.000293	−0.000322
1.8	−0.000159	−0.000157	−0.000143	−0.000154
2	0.000000	0.000000	0.000000	0.000000

## 8. Conclusions

This study employs the fractional three-phase-lag model to investigate generalized thermoelastic beams supported by a two-parameter elastic foundation. The key results are as follows:

▪ Different models (FLS, FTPL, and TPL with α=1) tend to forecast variations in the magnitudes of displacement, moment, and deflection, and frequently display reductions compared with the classical coupled thermoelastic (FCTE) model, with the exception of temperature (θ). This highlights the necessity of using more advanced thermoelastic theories, such as the fractional three-phase-lag (FTPL) model, which has a superior ability to observe heat wave scattering and unconventional heat transfer phenomena, such as ballistic transport, at the micro-scale.
▪ The mechanical response of a microbeam, including its displacement (u), moment (M) and deflection (w), is strongly influenced by the two-parameter elastic foundation $\;(K_{1},\;K_{2})$. Increasing these coefficients effectively reduces internal stresses and dynamic deformations, resulting in a faster and more stable beam setup. The study demonstrates that the temperature (θ) response remains largely independent of the elastic foundation coefficients.
▪ Introducing a fractional-order parameter (α) significantly alters the temperature (θ) distribution and mechanical responses. Lowering the fractional-order coefficient generally leads to higher temperature (θ) values and more pronounced thermal effects compared to standard integer-order models (α=1). The fractional-order coefficient (α) effectively mimics the memory and nonlocal effects inherent in advanced microstructures, providing a degree of freedom lacking in classical models.

## Figures and Tables

**Table 1:** Material and physical properties of the micro-scale beam.

Parameter	Symbol	Value	Units
Reference Temperature	$T_{0}$	293	K
Thermal Conductivity	K	156	$Wm^{-1}K^{-1}$
Modulus of Elasticity	E	169	GPa
Thermal-Expansion Coefficient	$\alpha_{t}$	$2.59\times 10^{-6}$	$K^{-1}$
Poisson’s Ratio	ν	0.22	-
Density	ρ	2330	$Kgm^{-3}$
Thermal Diffusivity	k	$9.4\times 10^{-5}$	$m^{2}s^{-1}$
Specific Heat	$C_{E}$	713	$\frac{J}{kgK}$
Reference Temperature	$T_{0}$	293	K
Thermal Conductivity	K	156	$Wm^{-1}K^{-1}$
Modulus of Elasticity	E	169	GPa

## Data Availability

The data are provided within the manuscript. Additional data are available from the corresponding authors upon request.
